# Regulation of an Autoimmune Model for Multiple Sclerosis in Th2-Biased GATA3 Transgenic Mice

**DOI:** 10.3390/ijms15021700

**Published:** 2014-01-23

**Authors:** Viromi Fernando, Seiichi Omura, Fumitaka Sato, Eiichiro Kawai, Nicholas E. Martinez, Sadie Faith Elliott, Keigyou Yoh, Satoru Takahashi, Ikuo Tsunoda

**Affiliations:** 1 Department of Microbiology and Immunology, Center for Tumor and Molecular Virology, Louisiana State University Health Sciences Center, Shreveport, LA 71130, USA; E-Mails: viromi.fernando@gmail.com (V.F.); omura.s@hotmail.com (S.O.); fsato@lsuhsc.edu (F.S.); ekawai-thk@umin.ac.jp (E.K.); nmarti@lsuhsc.edu (N.E.M.); Iloveham2011@yahoo.com (S.F.E.); 2 Department of Nephrology, Division of Clinical Medicine, Faculty of Medicine, University of Tsukuba, Tsukuba, Ibaraki 305-8575, Japan; E-Mail: k-yohnpr@umin.ac.jp; 3 Department of Anatomy and Embryology, Faculty of Medicine, International Institute for Integrative Sleep Medicine (WPI-IIIS), Life Science Center, Tsukuba Research Alliance (TARA), Laboratory Animal Resource Center (LARC), University of Tsukuba, 1-1-1 Tennoudai, Tsukuba, Ibaraki 305-8575, Japan; E-Mail: satoruta@md.tsukuba.ac.jp

**Keywords:** autoimmune demyelinating diseases, GATA3 transcription factor, autoimmunity, animal models, paraffin, histology, oligodendrocyte-myelin glycoprotein, Th1-Th2 assays, Luxol fast blue, Th17, incomplete Freund’s adjuvant

## Abstract

T helper (Th)2 cells have been proposed to play a neuroprotective role in multiple sclerosis (MS). This is mainly based on “loss-of-function” studies in an animal model for MS, experimental autoimmune encephalomyelitis (EAE), using blocking antibodies against Th2 related cytokines, and knockout mice lacking Th2-related molecules. We tested whether an increase of Th2 responses (“gain-of-function” approach) could alter EAE, the approach of novel GATA binding protein 3 (GATA3)-transgenic (tg) mice that overexpress GATA3, a transcription factor required for Th2 differentiation. In EAE induced with myelin oligodendrocyte glycoprotein (MOG)_35–55_ peptide, GATA3-tg mice had a significantly delayed onset of disease and a less severe maximum clinical score, compared with wild-type C57BL/6 mice. Histologically, GATA3-tg mice had decreased levels of meningitis and demyelination in the spinal cord, and anti-inflammatory cytokine profiles immunologically, however both groups developed similar levels of MOG-specific lymphoproliferative responses. During the early stage, we detected higher levels of interleukin (IL)-4 and IL-10, with MOG and mitogen stimulation of regional lymph node cells in GATA3-tg mice. During the late stage, only mitogen stimulation induced higher IL-4 and lower interferon-γ and IL-17 production in GATA3-tg mice. These results suggest that a preexisting bias toward a Th2 immune response may reduce the severity of inflammatory demyelinating diseases, including MS.

## 1. Introduction

Multiple sclerosis (MS) is an inflammatory demyelinating disease of the central nervous system (CNS). While the etiology of MS is yet to be determined, several factors have been associated with the initiation and exacerbation of MS, including anti-myelin autoimmunity, infectious agents, gender, environmental factors, and genetic background [1]. The autoimmune etiology of MS has been supported by the presence of autoantibodies and autoreactive T cells to myelin proteins, which have been found in MS patients, and the suppression of clinical signs of MS by immunomodulatory drugs [2–5].

Experimental autoimmune (or allergic) encephalomyelitis (EAE), an autoimmune model of MS, has also supported the autoimmune etiology of MS. EAE can be induced by sensitizing animals with CNS antigens, such as myelin oligodendrocyte glycoprotein (MOG), myelin proteolipid protein (PLP), and myelin basic protein (MBP), or their peptides [6,7]. Susceptibility to EAE depends on several factors, including the encephalitogenic antigen, and animal species and strain [8]. The genetic susceptibility to EAE has been associated with major histocompatibility complex (MHC), as well as non-MHC genes [9].

CD4^+^ T helper (Th) cells can be classified into Th1, Th2, or Th17 cell subsets. These subsets are classified based on the cytokines they secrete [10]. Th1 cells produce interleukin (IL)-2 and interferon (IFN)-γ, Th2 cells produce IL-4, IL-5, IL-6, IL-10, and IL-13. Th17 cells produce IL-17, IL-21, and IL-22. In MS and EAE, pro-inflammatory Th1 and Th17 cells have been associated with disease onset, progression, and relapse [11,12], while Th2 cells have been associated with disease remission [13]. Recovery from EAE has been correlated with increased levels of IL-4 and IL-10 mRNA within the CNS [14]. IL-4^−/−^ and IL-10^−/−^ mice have demonstrated increased susceptibility to EAE [15,16]. IL-10 could play a role in the regulation of EAE, as IL-10^−/−^ mice developed a severe non-remitting disease, while mice overexpressing IL-10 were protected [16,17].

In previous EAE experiments, the role of Th2 cells has generally been investigated by using gene knockout mice or blockade of Th2 cytokines by antibodies directed against Th2 cytokines and related molecules (“loss-of-function” approach) [17,18]. Although these “loss-of-function” studies were informative, it is unclear whether a differential increase (bias) in Th2 cells affects the susceptibility and/or the clinical course of EAE. Unlike conventional EAE models, there are several EAE models where a Th2 bias has been shown to lead disease progression [5,19–21], and the Th1 cytokine IFN-γ has been shown to play a protective role [22–27]. Importantly, “gain-of-function” mutations have been shown to alter Th immune responses and disease susceptibility to inflammatory diseases, including autoinflammatory diseases, in humans [28,29]. Thus, it is clinically relevant to test whether an increase in Th2 cells can affect the susceptibility to and disease course of EAE.

The master regulator of Th2 cells is the transcription factor, GATA binding protein 3 (GATA3). GATA3 is a member of the GATA family of zinc-finger transcription factors, which bind to the GATA consensus motif [30]. GATA3 is required for Th2 cell differentiation, stability, and cytokine production [31–34], and is almost exclusively expressed in T cells in adults [35]. We have established novel GATA3 transgenic (tg) mice that overexpress transcription factors. We have demonstrated that GATA3 mice have higher IL-4 and lower IFN-γ levels in their sera, compared with wild-type mice [36]. GATA3-tg mice have sensitivities to several immune-mediated disease models, compared with wild-type mice. GATA3-tg mice developed exacerbated allergen-induced airway inflammation and airway remodeling [37,38], as well as bleomycin-induced pulmonary fibrosis [30], while GATA3-tg mice had less severe autoimmune glomerulonephritis [36]. However, this novel transgenic mouse strain has never been used for CNS autoimmune diseases, including animal models for MS.

In this study, to test whether a bias to Th2 responses could alter EAE, we induced EAE in GATA3-tg mice. GATA3-tg mice had a significantly delayed onset of disease and a lower maximum clinical score, compared with wild-type mice. During remission, 83% of GATA3-tg mice recovered almost completely, while 60% of the wild-type mice showed incomplete recovery. Neuropathologically, GATA3-tg mice had decreased levels of meningitis and demyelination in the spinal cord, compared with wild-type mice. Immunologically, while both groups developed similar levels of MOG-specific lymphoproliferative responses, GATA3-tg mice had higher anti-inflammatory and lower pro-inflammatory cytokine profiles, compared with wild-type mice. Thus, a preexisting bias toward a Th2 immune response may reduce the severity of autoimmune demyelinating diseases, including MS.

## 2. Results and Discussion

### 2.1. Results

#### 2.1.1. Suppression of Clinical Disease in GATA3-tg Mice with EAE

**Figure 1.**
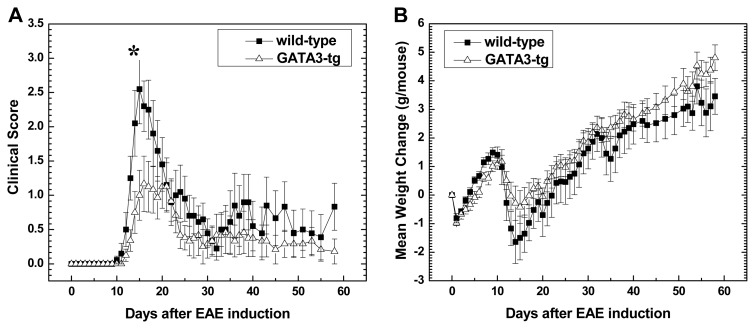
(**A**) Clinical score in wild-type and GATA3-tg mice sensitized with MOG_35–55_. Wild-type mice showed earlier disease onset and greater severity of disease than GATA3-tg mice. During the late stage, most GATA3-tg mice recovered completely, while wild-type mice did not; (**B**) GATA3-tg mice had less weight loss during disease onset, which mirrored the clinical signs. Values are mean ± SEM of nine wild-type and 11 GATA3-tg symptomatic mice. * *p* < 0.05. Representative of four experiments consisting of 58 mice in total.

We sensitized wild-type and GATA3-tg mice with MOG_35–55_, and then monitored the clinical signs and weight of the mice for two months. During the early stage of EAE (day 0 to 1 month post immunization (p.i.)), wild-type mice showed earlier disease onset (disease onset day p.i.: wild-type, 13.1 ± 0.3; GATA3-tg, 16.1 ± 0.3, *p* < 0.05) (Table 1) and greater severity of disease (wild-type, 3.4 ± 0.3; GATA3-tg, 2.1 ± 0.4, *p* < 0.05). However, the overall disease pattern and incidence was similar between wild-type and GATA3-tg mice (Figure 1A). MOG-sensitized wild-type mice typically developed severe hind limb paralysis or paraplegia at disease peak around day 15 p.i., followed by remission by one month p.i. (Table 1). On the other hand, MOG-sensitized GATA3-tg mice developed only mild tail or hind limb paresis, which was followed by remission by one month p.i. The amount of weight change had an inverse relationship to the severity of clinical signs. Wild-type mice tended to show more weight loss than GATA3-tg mice during the early stage (Figure 1B).

During the late stage (one to two months p.i.), most GATA3-tg mice recovered completely, while about 70% of wild-type mice showed incomplete recovery with neurological deficits (Table 1) [39]. There was also a difference in the incidence of relapse during the late stage, where we observed relapse in 30% and 8% of wild-type and GATA3-tg mice, respectively.

The cumulative clinical score showed that wild-type mice had greater morbidity than GATA3-tg mice during both the early and late stages (Table 1). There was no significant difference in incidence or onset of EAE between male and female mice within each strain, in the wild-type or GATA3-tg mice; when male and female EAE mice were evaluated separately within each strain, GATA3-tg mice showed less severe disease in either sex, compared with wild-type mice (data not shown).

#### 2.1.2. Suppression of Inflammatory Demyelination in GATA3-tg Mice

**Figure 2.**
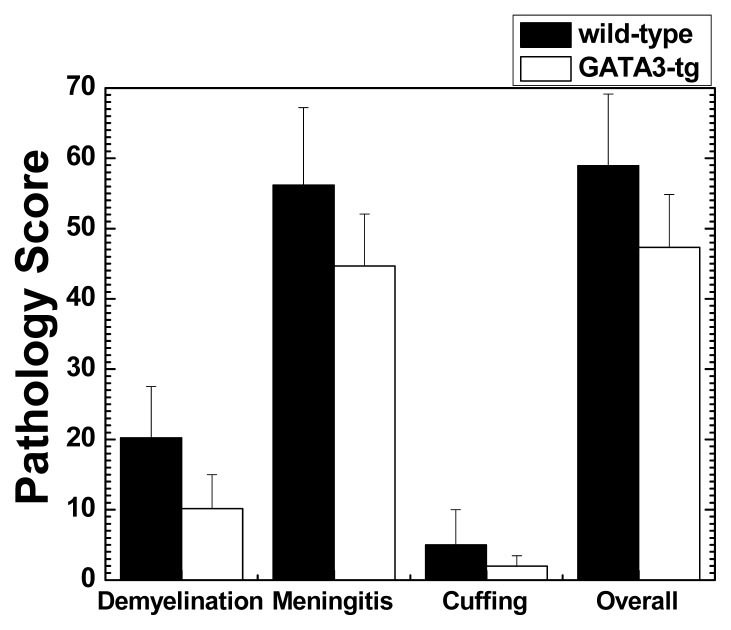
Spinal cord pathology of wild-type and GATA3-tg mice during the late stage of EAE. GATA3-tg mice (open column) had decreased pathology scores in meningitis, demyelination, and overall pathology, compared with wild-type mice (closed column). Values are representative mean pathology score + SEM of 10 wild-type and 12 GATA3-tg mice from four independent experiments.

**Figure 3.**
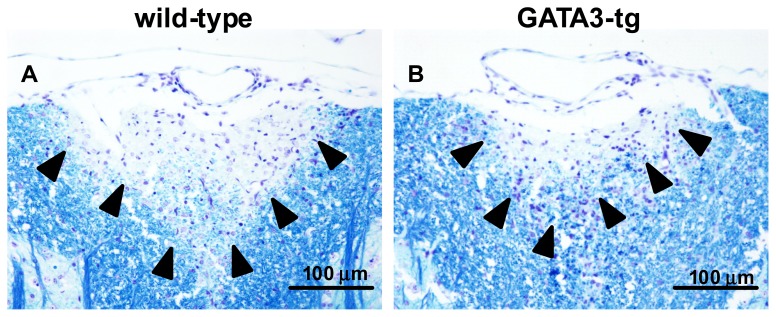
Demyelinating lesions in the spinal cord of wild-type (**A**) and GATA3-tg mice (**B**) during the late stage of EAE. Wild-type mice developed more severe demyelination, compared with GATA3-tg mice. Luxol fast blue stain. Magnification: ×171.

We examined whether GATA3-tg mice had differences in neuropathology compared with wild-type mice. We compared the inflammation and distribution of lesions in the CNS, two months p.i. between GATA3-tg and wild-type mice. Both GATA3-tg and wild-type mice developed inflammatory demyelinating lesions in the spinal cord, particularly, the dorsal funiculus. In the spinal cord, GATA3-tg mice had decreased levels of meningitis and demyelination, compared with wild-type mice, although they did not reach statistical differences (Figures 2 and 3). Both groups had only mild perivascular cuffing (inflammation). In general, the extent of inflammation and demyelination was well associated with clinical severity of disease (two months p.i., there was only a trend, but not statistical difference, of increased clinical signs as well as pathology scores in GATA3-tg mice compared with wild-type mice). There were no differences in the distribution (level of spinal cord segment) of the inflammatory demyelinating lesions, in which infiltrates were composed mainly of mononuclear cells (MNCs) with a few polymorphonuclear cells (PMNs, neutrophils and/or eosinophils), (PMN numbers per spinal cord segment: early stage wild-type, 4.9 ± 0.9; GATA3-tg, 3.1 ± 0.4 (*p* = 0.09); late stage wild-type, 0.2 ± 0.1; GATA3-tg, 0.2 ± 0.1, *p* = 0.75)). Thus, GATA3-tg mice had decreased overall levels of inflammatory demyelination in the spinal cord without alteration in the lesion distribution or PMN numbers in the infiltrates.

In the brain, there were no differences in pathology scores between the two mouse groups (pathology score: wild-type, 7.3 ± 1.3; GATA3-tg, 7.3 ± 1.2, *p* = 0.97). The brain lesions consisted of mild to moderate levels of perivascular cuffing and meningitis, while demyelination was rare. We also examined the histology of general organs in both groups of mice. While we found sporadic periportal lymphocyte infiltration in the liver in a few GATA3-tg mice, no obvious pathology was found in the kidney, thymus, or heart, in both groups (data not shown).

#### 2.1.3. No Change in T Cell Ratio among Infiltrates or Axonal Degeneration in GATA3-tg Mice

**Figure 4.**
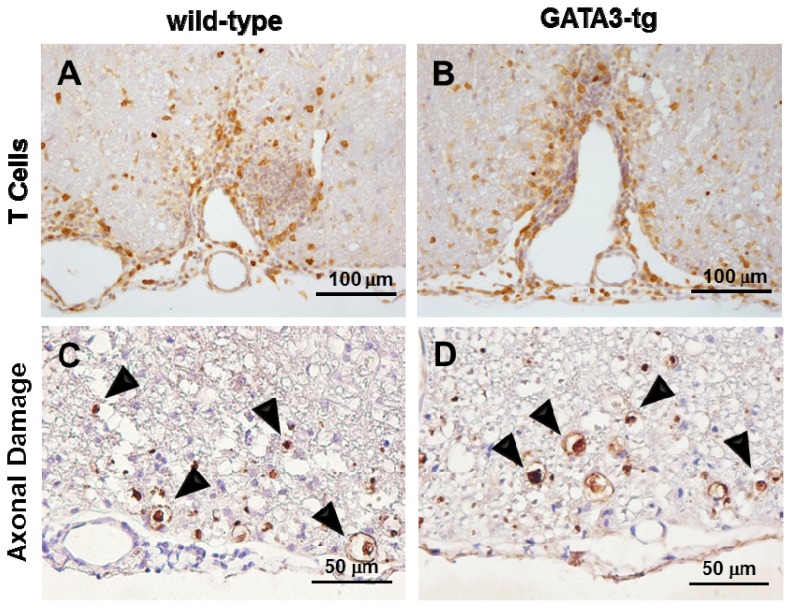
Similar percentages of CD3^+^ T cells among MNC infiltrates were observed in the spinal cord lesions of wild-type (**A**) and GATA3-tg mice (**B**) by anti-CD3 immunohistochemistry, two weeks p.i. (**A**,**B**). Similar numbers of damaged axons (arrowheads) were observed in wild-type (**C**) and GATA3-tg mice (**D**) by anti-nonphosphorylated neurofilament staining, two months p.i. (**C**,**D**). Representative spinal cord sections from five independent experiments. Magnification: (**A**,**B**) ×171; (**C**,**D**) ×342.

To further compare the components of cell infiltrates in the CNS, we conducted immunohistochemistry using anti-CD3 antibody and quantified the CD3^+^ T cells in meningeal cell infiltrates in the spinal cord during the early and late stages of EAE in both mouse groups (Figure 4A,B). There were no significant difference in the percentage of CD3^+^ T cells among MNC infiltrates between the two groups, while GATA3-tg mice tended to have higher percentages of CD3^+^ T cells among MNC infiltrates than wild-type mice in both the early and late stages (CD3^+^ T cells/total MNCs: early stage wild-type, 21.9% ± 10.7%; GATA3-tg, 31.9% ± 13.1% (*p* = 0.6); late stage wild-type, 11.3% ± 2.6%; GATA3-tg, 13.4% ± 2.2% (*p* = 0.5)). We also found no differences in the percentages of CD3^+^ T cells among cell infiltrates in the parenchyma or perivascular inflammation between the two groups of mice (data not shown).

We also compared the levels of axonal damage in the spinal cord between the two mouse groups, using immunohistochemistry against non-phosphorylated neurofilament protein (NFP), as described previously [40]. In both mouse groups, we detected a substantial number of damaged axons in the spinal cord white matter, particularly the ventral and ventrolateral funiculi (Figure 4C,D). The levels of axonal degeneration were similar during the early and late stages of EAE in both mouse strains. There were no significant differences between the groups in numbers of damaged axons per spinal cord quadrant: early stage wild-type, 11.3 ± 3.1; GATA3-tg, 6.5 ± 1.2 (*p* = 0.1); late stage wild-type, 12.5 ± 2.6; GATA3-tg, 19.9 ± 5.9 (*p* = 0.3).

#### 2.1.4. Altered Cytokine Profiles without Changes in the Overall MOG-Specific Lymphoproliferation in GATA3-tg Mice

**Figure 5.**
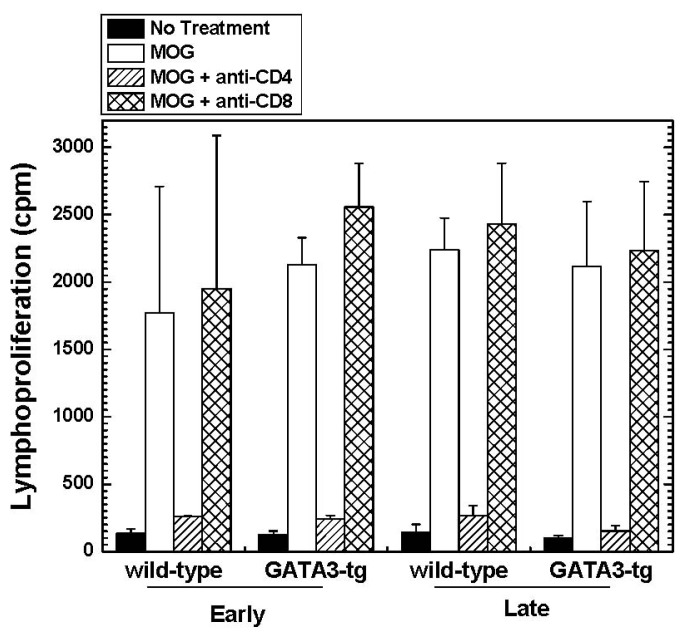
Lymphoproliferative responses of wild-type and GATA3-tg mice with EAE two weeks (early) and two months (late) p.i. MNCs were cultured with MOG_35–55_ in the presence or absence of anti-CD4 (hatched bars) or anti-CD8 (cross-hatched bars) antibody. Both groups of mice showed substantial MOG-specific proliferation with no significant differences between the groups. In both groups, anti-CD4 antibody incubation significantly reduced MOG-specific lymphoproliferation, whereas anti-CD8 antibody incubation had no effect. Lymph nodes were pooled from two to three mice. Values are mean + SEM of two to three pools of lymph node cells and are representative of one experiment (early) and four experiments (late) with similar results.

To test whether the levels of MOG-specific lymphoproliferative responses correlated with the clinical signs, we isolated MNCs from the regional lymph nodes (inguinal) of each group during the early and late stages of EAE, and conducted a lymphoproliferative assay stimulated with MOG_35–55_. We found similar levels of MOG-specific lymphoproliferative responses between wild-type and GATA3-tg mice (Figure 5). In both groups of mice, the MOG-specific lymphoproliferative responses were primarily mediated by CD4^+^ cells, but not CD8^+^ cells, as blockade of CD4^+^ T cells by incubation with anti-CD4 antibody, but not anti-CD8 antibody, inhibited MOG-specific proliferation [41]. There was no difference in [^3^H]thymidine incorporation in wells with no stimulation (spontaneous proliferation) between the mouse groups [42].

**Figure 6.**
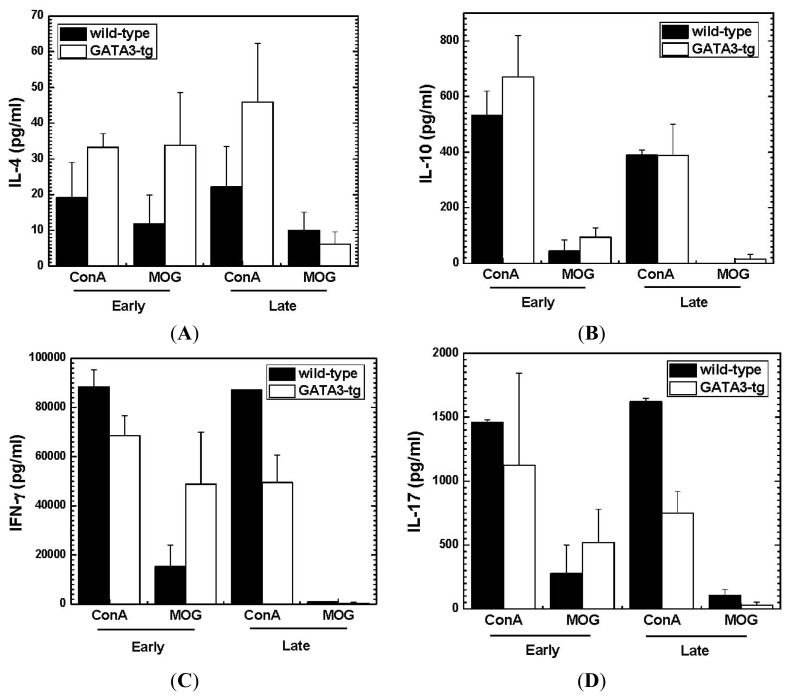
Cytokine profiles of wild-type and GATA3-tg mice with EAE. MNCs were stimulated with concanavalin A (ConA) or MOG. The levels of IL-4 (**A**); IL-10 (**B**); IFN-γ (**C**); and IL-17 (**D**) were measured by enzyme-linked immunosorbent assays (ELISA). During the early stage (two weeks p.i.), levels of IL-4 and IL-10 were higher in GATA3-tg mice after MOG or ConA stimulation. During the late stage (two months p.i.), MOG stimulation did not induce substantial cytokine production, while ConA stimulation induced higher IL-4, and lower IFN-γ and IL-17 in GATA3-tg mice. Cytokine assays were conducted in duplicate wells, using lymph nodes that were pooled from two to three mice. Values are mean + SEM of two to three pools of lymph node cells and are representative of two independent experiments (total numbers of samples: acute stage, 14 samples in six pools; chronic stage, 13 samples in six pools).

We also determined the cytokine profiles, Th2 cytokines (IL-4 and IL-10), Th1 cytokine (IFN-γ) and Th17 cytokine (IL-17A), using MNCs from the spleen and lymph node, and stimulated with MOG_35–55_ or a mitogen concanavalin A (ConA) (Figure 6). During the early stage, we detected higher levels of IL-4 and IL-10 in GATA3-tg mice with both MOG_35–55_ and ConA stimulation than in wild-type mice, while levels of pro-inflammatory IFN-γ and IL-17 tended to differ depending on stimuli. During the late stage, ConA stimulation induced higher IL-4, and lower IFN-γ and IL-17 in GATA3-tg mice, compared with wild-type mice, while MOG stimulation did not induce substantial amounts of any of the four cytokines in either mouse group.

### 2.2. Discussion

Using GATA3-tg mice, we demonstrated that a genetic background biased toward a Th2 immune response reduced Th1/Th17-mediated inflammatory demyelinating disease. While our current study demonstrated that the levels of GATA3, which were 10-fold higher in GATA3-tg mice than wild-type mice (Fold change: wild-type, 1.0 ± 0.2; GATA3-tg, 9.7 ± 0.8), a crucial factor for Th2 differentiation, directly affected the severity of EAE, other non-MHC genes, associated with Th2 immune responses, have previously been shown to influence susceptibility to EAE. For example, SJL/J mice are highly susceptible to several EAE models; SJL/J mice have been shown to be defective in their ability to produce IL-4 in the primary immune response to protein antigens [43,44]. This defect was related to a marked deficiency in natural killer T (NKT) cells [44], which have been shown to play a regulatory role in EAE and other MS models [15,45,46]. B10.S mice are genetically resistant to EAE; lymphocytes from B10.S mice did not generate IFN-γ in response to MBP, unless the cells were cultured *in vitro* in the presence of IL-12 [47], a cytokine that is known to promote differentiation to Th1 cells [48].

During the early stage of EAE, we harvested spleen MNCs from wild-type and GATA3-tg mice that had comparable EAE scores (score 2), a few days after disease onset rather than harvesting MNCs at disease peak to compare cytokine profiles between two mouse strains with similar neurological deficit. Despite similar levels of clinical scores, GATA3-tg mice produced higher amounts of IL-4 and IL-10, compared with wild-type mice. An altered cytokine profile in GATA3-tg mice may play a role in the delayed onset and reduced clinical severity during the early stage. Jäger *et al*. [49] investigated a role of Th2 cells in EAE by conducting adoptive transfer experiment, where MOG-specific CD4^+^ T cells were cultured in Th2 polarizing conditions. They found that only a few recipient mice developed EAE and that the EAE mice had a delayed onset of disease and reduced maximum clinical scores, which is similar to our current data shown in Table 1 and Figure 1. Interestingly, during disease onset, GATA3-tg mice tended to show increased MOG_35–55_-specific IFN-γ and IL-17 production (ConA induced the opposite effect), although there were no statistical differences, compared with wild-type mice (Figure 6). This suggested a higher clinical threshold to IFN-γ and IL-17 in GATA3-tg mice to induce comparable clinical signs in wild-type mice who had lower IL-4 and IL-10 levels, although this does not deny the possible protective role of IFN-γ [11] or other mechanisms [31].

Th17 cells have been shown to be involved in tissue damage (immunopathology) in immune-mediated diseases, although the precise pathomechanisms are unclear. In EAE, Th17 cells have been reported to play a role in the pathogenesis; for instance, IL-17^−/−^ mice had delayed onset of EAE and decreased severity of clinical signs [50–52]. Neutralization of IL-17 by injecting anti-IL-17 antibody into EAE mice ameliorated clinical signs [53]. Here, not only Th2 but also Th1 immune responses have been reported to be protective in EAE [23,54], as both Th1 and Th2 immune responses have been reported to inhibit Th17 immune responses by producing IFN-γ (Th1) and IL-4 (Th2) [55,56]. In this study, we showed that GATA3-tg mice had delayed onset of EAE and less clinical signs during the early stage of EAE, compared with wild-type mice. In MOG_35–55_ stimulation, during the early stage, GATA3-tg mice had larger amounts of IFN-γ and IL-4 production compared with wild-type mice.

During the late stage, GATA3-tg mice had higher IL-4, and lower IFN-γ and IL-17 production, compared with wild-type mice in response to ConA stimulation. This was associated with lower clinical (often complete remission and fewer relapses) and pathological scores (less severe meningitis and demyelination) in GATA3-tg mice, while about two thirds of wild-type mice had neurological deficit during the late stage. Interestingly, despite the change in cytokine profiles in GATA3-tg mice, GATA3-tg mice had comparable MOG-specific lymphoproliferative responses. One may assume that clinical signs of EAE and lymphoproliferative responses should correlate with each other, and thus, our results lead to controversy. However, this assumption is not necessarily true, as the total amount of lymphoproliferative responses are composed of not only those of proliferation of pro-inflammatory Th1 and Th17 cells, but also those of regulatory cells, including Th2 cells and other immune cell subsets.

Similarly, one may assume that the level of T cell infiltration in the CNS in EAE should correlate with clinical signs. Histologically, we found substantial CD3^+^ T cells in the spinal cords of GATA3-tg mice during the early and late stage of disease despite the mice having only mild clinical signs. One explanation could be that these CD3^+^ cells were anti-inflammatory Th2 cells. This would reconcile the discrepancy between clinical signs and levels of CD3^+^ T cells seen in the CNS. Indeed, CD3^+^ T cells have been shown to play a neuroprotective role, rather than cause immunopathology in the CNS. We have previously reported that the presence of CD3^+^ T cells in the CNS correlated with neuroprotection in CNS virus infection [57].

In this study, only a few GATA3-tg mice showed ataxia and most GATA3-tg mice developed classical EAE signs of tail and hind limb paralysis, although Th2 bias in EAE has been associated with ataxic EAE, which involves loss of balance, rolling and rotation [19]. Lees *et al.* [25] showed that CNS lesion location patterns were influenced by IFN-γ and its presence was selectively anti-inflammatory in the cerebellum and brainstem, which are the anatomical locations of lesions associated with ataxia. Elevated IFN-γ levels in GATA3-tg mice in the early stage may provide an explanation for a low incidence of ataxic form in GATA3-tg mice. In addition, the ataxic form of EAE has also been associated with anti-MOG antibody deposition in the CNS [5,19], and unlike whole MOG or MOG_92–106_-sensitized EAE, MOG_35–55_ sensitization did not induce strong antibody responses (data not shown).

Eosinophilic infiltration, has been reported in the CNS in human MS-like disease, atopic myelitis [58]. In asthma, Nakamura *et al*. [59] demonstrated that eosinophils were predominant in airway inflammation with concomitant significant GATA3 mRNA upregulation in asthmatic patients compared with healthy controls. In a murine model of asthma, GATA3-tg mice had more severe airway inflammation with eosinophilic infiltration in the lungs compared with wild-type mice [37,60]. However, GATA3-tg mice with EAE in our study had only minimal levels of CNS infiltration of PMNs, including eosinophils, comparable to wild-type mice.

In summary, we used a novel and innovative approach, GATA3-tg mice, to ascertain how a Th2-biased immune response would affect the onset, severity and/or progression of an autoimmune models for MS. Th2 bias ameliorated EAE clinically and pathologically, which was associated with changes in the cytokine profiles in GATA3-tg mice, while the overall levels of MOG-specific lymphoproliferative responses were similar between wild-type and GATA3-tg mice. Therefore, we have demonstrated that a preexisting bias towards Th2, immune responses influenced the susceptibility to an autoimmune model for MS. We believe that the data obtained from the current study are highly significant because they may lead to a future translational study of MS, by determining how a genetic bias to each Th subset is responsible for clinical and histological patterns of demyelinating diseases. When it is elucidated how Th bias from genetics or environmental factors can influence MS clinically and histologically, this information can be used clinically among MS patients and their family members, as a means of personalized medicine in prediction of disease courses (e.g., disease progression or remission) or determination of treatment (e.g., responses to therapies may depend on preexisting Th balance) [61].

## 3. Experimental Section

### 3.1. EAE Induction

To generate GATA3-tg mice, 2.0-kb murine, full-length GATA3 cDNA was inserted into the VA vector and injected into BDF/fertilized eggs [36]. The VA vector contains a CD2 transgenic cassette including the upstream gene regulatory region and locus control region of the human *CD2* gene [62]. GATA3 transgene has preferential expression in T cells [60]. We sensitized 6-week-old GATA3-tg and C57BL/6 wild-type mice (Harlan Laboratories, Inc., Indianapolis, IN, USA) subcutaneously in the base of the tail with 100 nmol of MOG_35–55_ peptide (United Peptide Corporation, Rockville, MD, USA) emulsified in complete Freund’s adjuvant composed of Imject^®^ Freund’s incomplete adjuvant (Pierce Biotechnology, Rockford, IL, USA) and *Mycobacterium tuberculosis* H37 Ra (Difco Laboratories, Detroit, MI, USA) [39]. The final concentration of *M. tuberculosis* in the MOG/complete Freund’s adjuvant solution was 2 mg/mL (200 μL/mouse). Mice were also injected intraperitoneally with 400 ng of pertussis toxin (List Biological Laboratories, Inc., Campbell, CA, USA) on days 0 and 2 after MOG sensitization [39].

Clinical scores of EAE were evaluated as follows: 0, no signs; 1, paralyzed tail; 2, mild hind limb paresis; 3, moderate hind limb paralysis; 4, complete hind limb paraplegia; 5, quadriplegia or moribund [20]. We assessed the clinical course of EAE mice during the early (day 0 to 1 month p.i.) and late stages (1 to 2 months p.i.), using mean maximum EAE scores and the cumulative score. The cumulative score was measured by the area under the EAE score graph, reflecting overall disease severity and morbidity over the designated time period [63].

### 3.2. Pathology

Mice were killed using isoflurane (VEDCO Inc., St. Joseph, MO, USA), 2 weeks or 2 months p.i. [20]. Mice were perfused with phosphate-buffered saline (PBS) followed by a 4% paraformaldehyde solution (Sigma-Aldrich, St. Louis, MO, USA) in PBS. The brain, spinal cord, and general organs were harvested and fixed with 4% paraformaldehyde. The spinal cord and brain were divided into 10 to 12 transverse segments and five coronal slabs, respectively, and embedded in paraffin. Four-μm-thick sections of CNS tissues were stained with Luxol fast blue (Solvent blue 38; Sigma-Aldrich) for myelin visualization. Sections of general organs and CNS sections for neutrophil and eosinophil counts were also stained with hematoxylin and eosin (Electron Microscopy Sciences, Hatfield, PA, USA).

For scoring of spinal cord sections, each spinal cord section was divided into four quadrants: the ventral funiculus, the dorsal funiculus, and each lateral funiculus [5]. Neuropathology was scored, in a blinded fashion; any quadrant containing demyelination, meningitis, or perivascular cuffing was given a score of 1 in that pathological class. The total number of positive quadrants for each pathological class was determined and then divided by the total number of quadrants present on the slide and multiplied by 100 to give the percent involvement for each pathological class. An overall pathology score was also determined by giving a positive score if any pathology was present in the quadrant, and presented as the percent involvement. Brain pathology scores were evaluated as follows: meningitis (0, no meningitis; 1, mild cellular infiltration; 2, moderate cellular infiltration; 3, severe cellular infiltration), perivascular cuffing (0, no cuffing; 1, 1 to 10 lesions; 2, 11 to 20 lesions; 3, 21 to 30 lesions; 4, 31 to 40 lesions; 5, over 40 lesions), and demyelination (0, no demyelination; 1, mild demyelination; 2, moderate demyelination; 3, severe demyelination). Each score from the brain was combined for a maximum score of 11 per mouse.

### 3.3. Immunohistochemistry

We visualized T cells and damaged axons by the avidin-biotin peroxidase complex (ABC) technique (Vectastain^®^ Elite ABC Kit, Vector Laboratories, Inc., Burlingame, CA, USA), using rabbit anti-CD3 antibody (1:32 dilution; DAKO Corporation, Carpinteria, CA, USA) and with SMI 311 (Sternberger Monoclonal, Inc., Baltimore, MD, USA), a cocktail of antibodies (SMI 32, 33, 37, 38, and 39; personal communication with Dr. Ludwig Sternberger) to nonphosphorylated NFP, respectively [40]. For antigen retrieval for CD3 or NFP immunostain, we treated sections with Vector^®^ Antigen Unmasking Solutions (Vector Laboratories, Inc., Burlingame, CA, USA) for 25 min or with distilled H_2_O for 15 min at 120 °C in the Digital Decloaking Chamber I (Biocare Medical, Concord, CA, USA), respectively [64]. The numbers of CD3^+^ T cells or SMI311^+^ damaged axons were counted under a light microscope using 10 to 12 transverse spinal cord segments per mouse as previously described [64].

### 3.4. Lymphoproliferative Assay

MNCs were isolated from the inguinal lymph nodes of EAE mice using Histopaque 1038 (Sigma-Aldrich) [20]. MNCs were cultured in RPMI 1640 medium (Mediatech Inc., Manassas, VA, USA), supplemented with 10% fetal bovine serum (FBS) (Mediatech), 2 mM l-glutamine (Mediatech), 50 mM β-mercaptoethanol (Sigma-Aldrich), and 1% antibiotic-antimycotic solution (Mediatech), at 2 × 10^5^ cells/well in 96-well plates (Corning, Inc., Corning, NY, USA). Cells were stimulated with 50 μg/mL MOG_35–55_ peptide for 5 days in the presence or absence of blocking antibody against CD4 (GK 1.5, American Type Culture Collection (ATCC), Rockville, MD, USA) or CD8 (Lyt 2.43, ATCC) [65]. To assess the lymphoproliferation, [^3^H]thymidine (PerkinElmer, Inc., Waltham, MA, USA) was added to the culture at the concentration of 1 μCi/well for the last 24 h. MNCs were harvested on Reeves Angel 934AH filters (Brandel, Gaithersburg, MD, USA), using PHD™ Harvester (Brandel). The incorporated radioactivity was measured by Wallac 1409 Liquid Scintillation Counter (PerkinElmer). All cultures were performed in triplicate.

### 3.5. Cytokine Assay

MNCs isolated from the spleens of EAE mice were cultured at 2 × 10^6^ cells/well in 96-well plates (Corning) and stimulated with 5 μg/mL of ConA or with MOG_35–55_ (final concentration 50 μg/mL) for 48 h [5]. Culture supernatants were harvested and stored at −80 °C until examined. The levels of IL-4, IL-10, IFN-γ (BD Biosciences, San Diego, CA, USA), and IL-17A (Biolegend, Inc., San Diego, CA, USA) production in culture supernatants were assessed by ELISA, according to the manufacturer’s instructions.

### 3.6. Statistical Analysis

Significant differences in EAE scores, including the cumulative EAE score (area under the EAE score curve), between GATA3-tg mice and controls were assessed using Mann Whitney *U*-test. Student *t*-test was used to determine the significance level in the other experiments, using OriginPro 8.1 (OriginLab Corporation, Northampton, MA, USA). Using five spinal cords per group, we calculated CD3^+^ T cell percentages; the numbers of CD3^+^ T cells were divided by the total numbers of infiltrating MNCs counterstained by hematoxylin in, at least, five inflammatory areas of one spinal cord immunostained by anti-CD3 antibody.

## 4. Conclusions

In conclusion, the current study demonstrated that GATA3 overexpression (Th2 biased) can decrease disease severity and delay disease onset of EAE, an animal model for MS. The current results may lead to a future translational study of MS, by determining how a genetic bias to each Th subset is responsible for clinical and histological patterns of demyelinating diseases.

## Figures and Tables

**Table 1.** Clinical signs of experimental autoimmune encephalomyelitis (EAE) in wild-type and GATA3-tg mice a.

Mouse Strain	*N*	Onset Days	Maximum Clinical Score	Cumulative Score		Incidence	

				Early b	Late c	Early b	Late c
wild-type	10	13.1 ± 0.3	3.4 ± 0.3	23.9 ± 5.0	16.8 ± 7.9	9/10	6/10
GATA3-tg	12	16.1 ± 0.3 *	2.1 ± 0.4 *	12.8 ± 3.9	8.9 ± 6.0 **	11/12	3/12

a EAE were induced with myelin oligodendrocyte glycoprotein (MOG)_35–55_ peptide;

b Early stage, day 0 to 1 month post immunization (p.i.);

c Late stage, one to two months p.i.

* *p* < 0.05,

** *p* < 0.01 compared with wild-type. Shown are the mean ± SEM of symptomatic mice. Data are results from one experiment, which are representative of five experiments. *N*; mouse number sensitized with MOG_35–55_.
